# Optimization and Characterization of a Liposomal Azithromycin Formulation for Alternative Macrophage Activation

**DOI:** 10.3389/fddev.2022.908709

**Published:** 2022-07-05

**Authors:** Abdullah A. Masud, Fahd M. Alsharif, Jarrod W. Creameans, Jasmine Perdeh, David J. Feola, Vincent J. Venditto

**Affiliations:** 1 Department of Pharmaceutical Sciences, College of Pharmacy, University of Kentucky, Lexington, KY, United States; 2 Department of Pharmacy Practice and Science, College of Pharmacy, University of Kentucky, Lexington, KY, United States

**Keywords:** Azithromycin, drug delivery, immune modulation, liposome, macrophage

## Abstract

Liposomal azithromycin (L-AZM) promotes macrophage polarization toward an M2-like phenotype in the context of myocardial infarction that results in improved cardiovascular outcomes in mice. To improve upon this formulation, we sought to identify optimized formulation, stability, and biological activity parameters necessary to enhance the immunomodulatory activity and efficacy of L-AZM. While our parent formulation contains a mixture of long-chain saturated phosphatidylcholine and phosphatidylglycerol lipids, we evaluated a series of formulations with different amounts of unsaturated lipids and cholesterol with the goal of improving the loading capacity and stability of the formulations. We also introduce fusogenic lipids to improve the cytosolic delivery to enhance the immune modulatory properties of the drug. To achieve these goals, we initially prepared a library of 24 formulations using thin film hydration and assessed the resultant liposomes for size and polydispersity. Five lead formulations were identified based on low polydispersity (<0.3) and stability over time. The lead formulations were then evaluated for stability in serum using dialysis and macrophage polarization activity *in vitro* as measured by decreased IL-12 expression. Collectively, our data indicate that the formulation components drive the balance between encapsulation efficiency and stability and that all the lead liposomal formulations improve *in vitro* alternative macrophage activation as compared to free AZM.

## INTRODUCTION

Myocardial infarction (MI) and its ensuing damage is the leading cause of death in developed nations, affecting over 3 million patients annually (Force Members et al., 2012). Recurrent ischemia following MI often evolves into congestive heart failure, the most powerful predictor of mortality in patients with MI (Granger et al., 2003). Shortly after MI, hypoxia causes a large pro-inflammatory response accompanied by neutrophil infiltration into the ischemic myocardium to trigger apoptosis and clear cellular debris—a process that contributes to tissue damage (van der Laan et al., 2012; Swirski and Nahrendorf, 2018). After the initial inflammatory phase, macrophages and monocytes promote myocardial repair in a process that results in increased fibrosis and cardiac tissue scarring (Yan et al., 2013). Although many treatment options exist to diminish the progression of cardiac damage after MI, there are currently no approved therapies to modulate the immune response in this setting. Many drugs have shown less favorable results in clinical trials due to their immunosuppressive effects (Bulkley and Roberts, 1974; Schjerning Olsen et al., 2011), while others such as colchicine have a low therapeutic index and have recently shown varying results (Tardif et al., 2019; Tong et al., 2020). Therefore, new treatment paradigms are critical to reduce the burden of cardiovascular complications and improve post-MI outcomes in patients.

Azithromycin (AZM) is used primarily for its antimicrobial properties (Abtahi-Naeini et al., 2021; Bogdanov et al., 2021) but has more recently been evaluated for its anti-inflammatory and immunomodulatory effects (Equi et al., 2002; Clement et al., 2006; Saiman et al., 2010; Ratjen et al., 2012; Nichols et al., 2020; Venditto et al., 2021). These properties stem from the ability of AZM to alternatively polarize macrophages, shifting M1-like macrophages which are pro-inflammatory to a more reparative, anti-inflammatory M2-like phenotype (Murphy et al., 2008; Vrančić et al., 2012; Cory et al., 2013; Amantea et al., 2016; Haydar et al., 2019). The ability of AZM to polarize macrophages toward an M2-like phenotype in animals and humans and improve disease outcomes has been demonstrated in the context of ischemic stroke (Amantea et al., 2016), chronic obstructive pulmonary disease (Hodge et al., 2008), cystic fibrosis (Meyer et al., 2009), and more recently MI (Al-Darraji et al., 2018; Al-Darraji et al., 2020). An obstacle to the use of AZM in the context of MI, however, is the incidence of cardiac arrythmias observed in human studies, which has resulted in FDA warnings for AZM and other macrolides in subjects with a history of cardiovascular complications (Giudicessi and Ackerman, 2013). In fact, our previous work with AZM in a murine model of MI shows increased mortality after intravenous administration of free AZM, underscoring the need for an improved delivery method (Al-Darraji et al., 2020).

Lipid nanoparticles used for drug delivery to macrophages provides a unique opportunity to counteract inflammatory damage associated with MI, while reducing exposure of cardiomyocytes to AZM. Some drug delivery strategies target the inflamed tissue directly using liposomes with polyethylene glycol (PEG) on their surface to prevent macrophage uptake in the blood and promote tissue accumulation (Torchilin et al., 1996; Levchenko et al., 2012; Dasa et al., 2015). However, by harnessing the phagocytic nature of immune cells trafficking to the injury, non-PEGylated liposomes are preferentially taken up en route to inflamed tissue, thereby decreasing cardiomyocyte uptake and reducing the risk of cardiotoxicity. Previous work by our group has shown that liposomal AZM (L-AZM) succeeds in targeting immune cells over cardiomyocytes, thereby significantly reducing the cardiotoxicity relative to free AZM (Al-Darraji et al., 2020). Additionally, in a murine model of cardiac ischemia, L-AZM significantly decreases mortality as compared to those treated with an equivalent dose of free AZM or vehicle control (Al-Darraji et al., 2020). Notably, free AZM led to reduced heart rate, arrhythmia, and sudden cardiac death, which was not observed using the same dose of AZM encapsulated in a liposome. These preclinical data support continued investigation of liposomal delivery as a therapeutic strategy to reduce the burden of post-MI inflammation in patients with the goal of long-term event-free survival and improved long-term cardiac output.

The efficacy of our primary L-AZM formulation in lowering post-MI mortality in mice prompted the evaluation of additional formulations for further optimization. Since the parent formulation contains a mixture of long-chain saturated phosphatidylcholine (PC) and phosphatidylglycerol (PG) lipids, we hypothesized that the inclusion of unsaturated and fusogenic lipids would improve the stability and cytosolic delivery of the formulations. Unsaturated phospholipids have shown better efficiency in encapsulating both hydrophilic (Sakai et al., 2008) and lipophilic drugs (Pereira et al., 2016) compared to their saturated counterparts. A library of liposomal formulations was prepared and characterized, of which five lead formulations were selected based on size and polydispersity index for further analysis of encapsulation efficiency, stability and polarization activity. Herein, we describe the characterization and *in vitro* functional evaluation of the five lead L-AZM formulations with the goal of identifying an optimized formulation for continued *in vitro* and *in vivo* investigation.

## MATERIALS AND METHODS

### Reagents

Lipids were purchased from Avanti Polar Lipids (Alabaster, AL, United States) including 1,2-Distearoyl-sn-glycero-3-phosphocholine (DSPC, 25.0 mg/ml in chloroform), 1,2-Dioleoyl-sn-glycero-3-phosphocholine (DOPC, 25.0 mg/ml in chloroform), 1-Palmitoyl-2-oleoyl-glycero-3-phosphocholine (POPC, 25.0 mg/ml in chloroform), and 1,2-Distearoyl-sn-glycero-3-phospho-1′-rac-glycerol, sodium salt (DSPG). Powdered DSPG was dissolved in the solvent mixture of chloroform, methanol, and DI water at a ratio of 13:7:1, respectively, and made fresh at 25.0 mg/ml when required. Cholesterol was re-crystallized in ethanol before being dissolved in chloroform for use in formulations. LC-MS grade acetonitrile and methanol were procured from J.T. Baker (Philipsburg, NJ, United States). Azithromycin dihydrate was purchased from TCI. Monobasic potassium dihydrogen phosphate (KH_2_PO_4_) was purchased from VWR and manufactured by Amresco LLC (Dallas, TX, United States). DMEM (Dulbecco’s Modification of Eagle’s Medium with 4.5 g/L glucose, L-glutamine and sodium pyruvate), fetal bovine serum (FBS) and stable cell trypsin was procured from Corning (Corning, NY, United States).

### Liposomal Formulations

All liposomal formulations were prepared by thin film hydration according to an established protocol (Nardo et al., 2021). Briefly, phospholipids and cholesterol solutions were mixed in a round bottom glass tube at the desired molar ratio. Next, AZM dissolved in ethanol was added to the lipid mixture at 10 or 30 mol% of the total phospholipid content. Subsequently, solvents from the lipid-AZM mixture were evaporated using a rotary evaporator at 60°C to obtain a thin film layer on the glass tube surface, which was dried overnight under vacuum. The resulting thin film was rehydrated in PBS pH 7.5 and sonicated for 1 h at 60°C. Each formulation was prepared at a final lipid concentration of 40 mM. Empty liposome controls without AZM were also prepared for each formulation using the identical procedure.

### Formulation Size and Zeta Potential Measurement

The mean diameter, polydispersity index (PDI), and zeta potential of each formulation were measured on a Zetasizer Nano ZS (Malvern Panalytical, Malvern, United Kingdom). Liposomes were diluted 20-fold in hydration buffer, and all measurements were conducted at 25°C. During the size (mean diameter) and polydispersity measurements, ~1 ml of liposomes was placed in the regular plastic cuvettes, and the number of photons scattered by the liposome particles was measured at the detection angle of 175°. The mean diameter and PDI of the liposome particles were then estimated by photon correction spectroscopy. The zeta potential of the liposomes was measured by adding ~1 ml of the same 20-fold diluted liposomes to a folded capillary cell (DTS 1070). Zeta potential of the liposomes are correlated with fluctuations in intensity of the incident light scattered from the mobile liposome particles under applied voltage. As liposomes move through the capillary cell with applied voltage, light scattered by the particles is detected at 12.8° and all experiments are conducted at 25°C. Each reported zeta potential value is the average of 10 repeated measures.

### HPLC Method to Quantify Free Azithromycin

Quantification of released AZM was determined with Agilent 1,100 and Dionex ultimate 3,000 HPLCs equipped with a quaternary pump, diode array detector, and an autosampler. HPLC conditions were adapted from a published protocol with slight modification (Rukavina et al., 2018). In brief, separation was carried out using a C_18_ column (ACE equivalence, 100 mm × 4.6 mm, 5 μm) and the mobile phase consisting of 20 mM KH_2_PO_4_ aqueous solution (pH adjusted to 7.5) and acetonitrile at a 30:70 ratio. The mobile phase flow rate was 1.0 ml/min, and AZM was detected at the wavelength of 210 nm. Importantly, AZM bound to serum proteins (in experiments using FBS) and to dialysis chamber surfaces are not included in this measurement. A calibration curve was constructed using the AZM concentration ranging within 10–500 μg/ml. Under these conditions with our system, AZM eluted with a retention time of ~5 min.

### Determination of Encapsulation Efficiency

Un-encapsulated AZM was separated from the L-AZM by filtering the liposomes through a Sephadex G-25 mini-column, gravimetrically. In brief, 2.5 ml of liposome solution was eluted through the mini-column with 3.6–4.0 ml of PBS pH 7.5. To assess the concentration of AZM retained in the formulation after size exclusion column, 50 μL of liposomes were mixed with 950 μL of methanol. AZM content of each formulation was determined by HPLC before and after passing through the mini-column and the encapsulation efficiency of the liposomes from each formulation was calculated as the ratio of AZM content before and after filtration.

### Serum Stability of Liposomes and *in vitro* Azithromycin Release

Release kinetics of the AZM from all liposomal formulations were evaluated at physiological pH (PBS pH 7.5) in the presence and absence of 50% FBS. Liposomes were dialyzed using a snakeskin dialysis membrane (molecular weight cut-off: 10 kDa) with constant stirring at 60 rpm and maintaining the temperature at 37°C. Liposomes filtered through the Sephadex G-25 mini-column (1.0 ml) were mixed in a 1:1 ratio with PBS or FBS to achieve serum concentrations of 0% and 50% (mimicking human blood), respectively. The resulting mixtures were placed inside the snakeskin dialysis bag and dialyzed against 50 ml PBS in the outer media. The experimental design using 50% FBS in the dialysis tubing and PBS on the outside is based on previous reports and aligns with the goal of investigating the role of protein disruption of the liposomal bilayer, which are retained by the dialysis membrane (Jafari et al., 1998; Qin et al., 2011). AZM content in outer media was determined overtime at 1, 2, 4, 6, 8, and 24 h. During every sampling point, 1 ml of the outer media was aliquoted and re-compensated with fresh PBS pH 7.5. Free AZM was also evaluated to quantify dialysis kinetics for un-encapsulated AZM. All samples collected during the release studies were analyzed for AZM content using the HPLC conditions described previously. Results were quantified using a calibration curve for AZM with concentrations ranging from 2 to 70 μg/ml.

### Macrophage Polarization

In order to study the immune modulatory effects of L-AZM, *in vitro* polarization assays using J774 murine macrophages were performed. Prior to the assays, the cells were allowed to grow until they reached confluency in DMEM with 10% FBS and 1% penicillin/streptomycin. Once confluent, the cells were scraped, counted, and then plated at a density of 2.5 × 10^5^ cells per 1 ml of media in 24-well plates. The plated cells were then treated with interferon (IFN)-γ (final concentration 20 ng/ml) or a combination of IL-4 and IL-13 (final concentration 10 ng/ml each). Selected wells with IFNγ were treated with F-AZM (final concentration of 30 μM), or an L-AZM formulation (at approximately 30 μM AZM based on encapsulation efficiency). Cells were incubated for 6 h at 37°C in 5% CO_2_ before being stimulated with lipopolysaccharide (LPS) (final concentration 100 ng/ml) and incubated for 24 h. The cells were then centrifuged at 1,200 × *g* and the media supernatants collected for cytokine analysis. Interleukin (IL)-12 concentrations were measured using a sandwich ELISA kit according to manufacturer’s instructions (BioLegend, San Diego, California, United States).

### Statistical Analysis

Statistical analysis was performed using GraphPad Prism (GraphPad Software, La Jolla, CA). Comparison between groups was made via one-way ANOVA with Tukey’s test for multiple comparisons, paired sample T-test with McNemar’s test, or via two-way ANOVA with Sidak’s multiple comparisons test where appropriate. The *p*-value cut-off for statistical analysis was *p = 0.05, **p = 0.01, ***p = 0.001. Comparison of release rates were performed by calculating the difference factors (*f*_1_) and similarity factors (*f*_2_) for each formulation relative to free AZM and the other liposomal formulations, based on industry guidance from the FDA (US Department of Health and Human Services, 1997). Release rates are considered to be similar when 0 < *f*_1_ < 15 and when 50 < *f*_2_ < 100. Given that both equations indicate similarity in the same formulations only *f*_2_ is presented in the main text with *f*_1_ presented in the supporting information.

## RESULTS

Our goal of identifying a lead formulation for continued preclinical evaluation is based on a library of formulations containing AZM and preliminary evidence of stability as determined by low polydispersity index over a period of 10 days after formulation. The library was generated using an iterative approach to evaluate the effect of distinct changes to the formulation before completing a more thorough analysis of a series of 12 formulations prepared at two different lipid concentrations for a total of 24 formulations (Supplementary Table S1). From this library, five lead formulation containing AZM, and their empty counterparts, were prepared for further characterization (Table 1). All formulations were prepared by thin film hydration with AZM included in the thin film for incorporation in the bilayer during hydration. Notably, each formulation increased in size with the inclusion of 10 mol% AZM as compared to the empty formulations lacking drug, while the PDI and zeta potentials of each remained relatively unchanged. Each of the lead formulations containing AZM result in liposomes with mean diameters of 70–110 nm and low PDI (0.09–0.23). The negative charge of each formulation (~−25 mV) is due to the inclusion of DSPG as an anionic lipid in the formulations, which can improve AZM incorporation through ion pairing in the lipid bilayers (Matschiner et al., 1995; Ren et al., 2018).

Formulation 1 (F1) is based on a previously reported formulation investigated as an antimicrobial agent (Oh et al., 1995), and served as the formulation used to generate our previous *in vivo* data in a murine model of cardiac ischemia (Al-Darraji et al., 2020). F1 contains solely saturated lipids which results in slightly larger diameter with AZM as compared to formulations containing primarily unsaturated lipids. The size and PDI of F1 are also not altered with the inclusion of 25% DOPE as demonstrated with F5. Each of the other formulations containing unsaturated lipids (F2, F3, and F4) also exhibit low polydispersity and maintain their zeta potential in the presence or absence of AZM.

**FIGURE 1 |:**
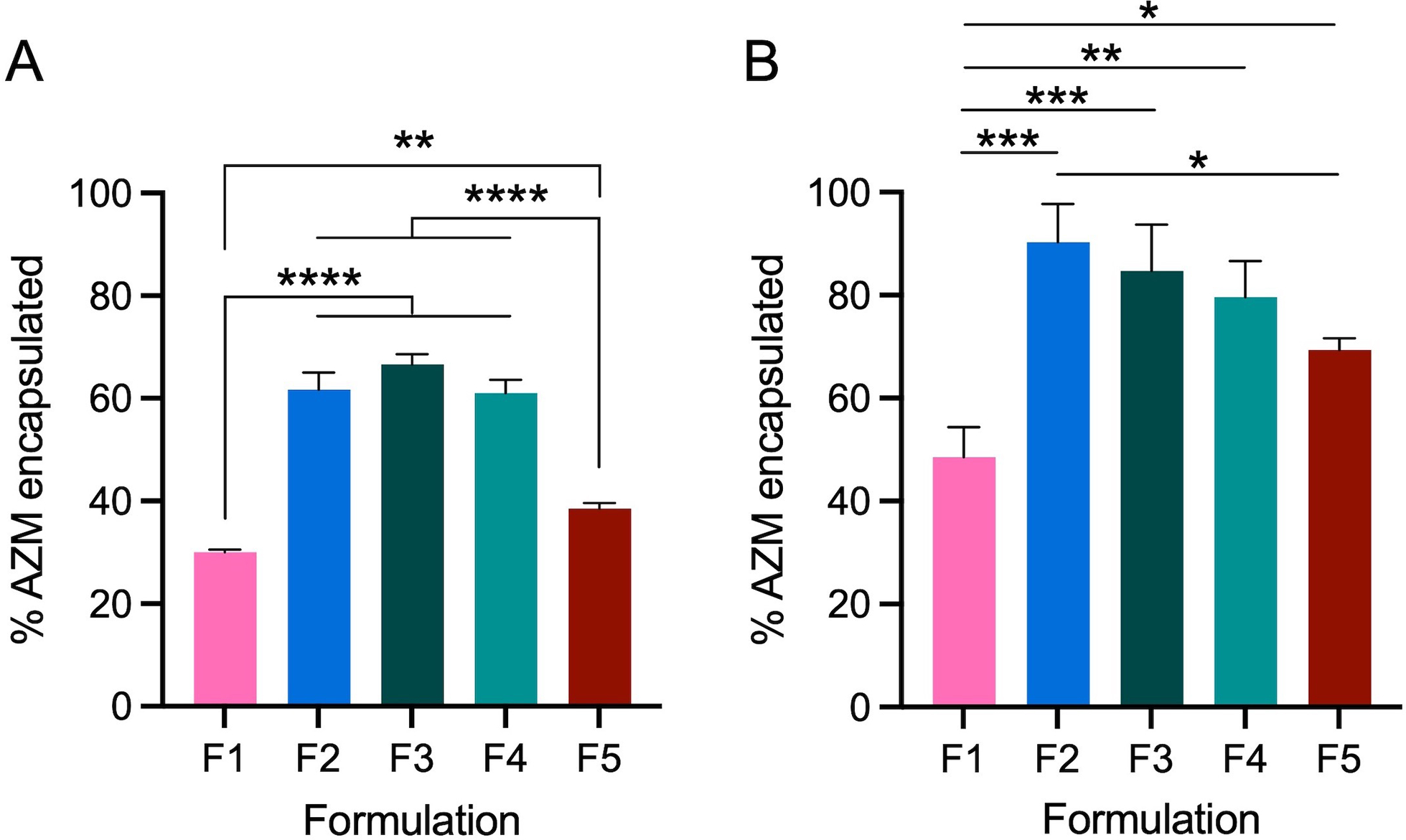
Liposomal encapsulation efficiency of AZM in five lead formulations as determined by HPLC. Baseline AZM encapsulation in each formulation is compared to the AZM content after removal of free drug by size exclusion chromatography. All formulations were prepared at 40 mM at constant volume with AZM added such that drug accounted for **(A)** 10 mol% or **(B)** 30 mol% relative to phospholipid content. Bars denote the mean ± SD (*n* = 3). Statistical analysis performed by ANOVA *p < 0.05; **p < 0.01; ***p < 0.001; ****p < 0.0001.

AZM encapsulation efficiency (EE%) was then quantified by HPLC for each of the formulations prepared with 10 and 30 mol% AZM relative to phospholipid content (Figure 1). Formulations were assessed for their incorporation of AZM immediately after size exclusion chromatography to remove free AZM, using a preelution sample to quantify total potential AZM incorporation. Notably, formulations containing more than 50% saturated lipids (F1 and F5) exhibit the lowest AZM encapsulation efficiencies with 30% and 39% respectively. Exchange of DSPC in F1 for POPC in F2 results in a 2-fold increase in EE% and AZM incorporation, which is similar to the other formulations with unsaturated lipids (F2 = 62%, F3 = 67%, F4 = 61%). When 30 mol % AZM is used in the formulations, the EE% improve by ~10–30% for each formulation (Figure 1B), and the general trend persists with F2, F3, and F4 achieving higher encapsulation efficiencies immediately after hydration. Using both concentrations of AZM, F1 remains the worst formulation for encapsulation efficiency and the cause for increased EE% with higher concentrations of AZM remains unclear.

**FIGURE 2 |:**
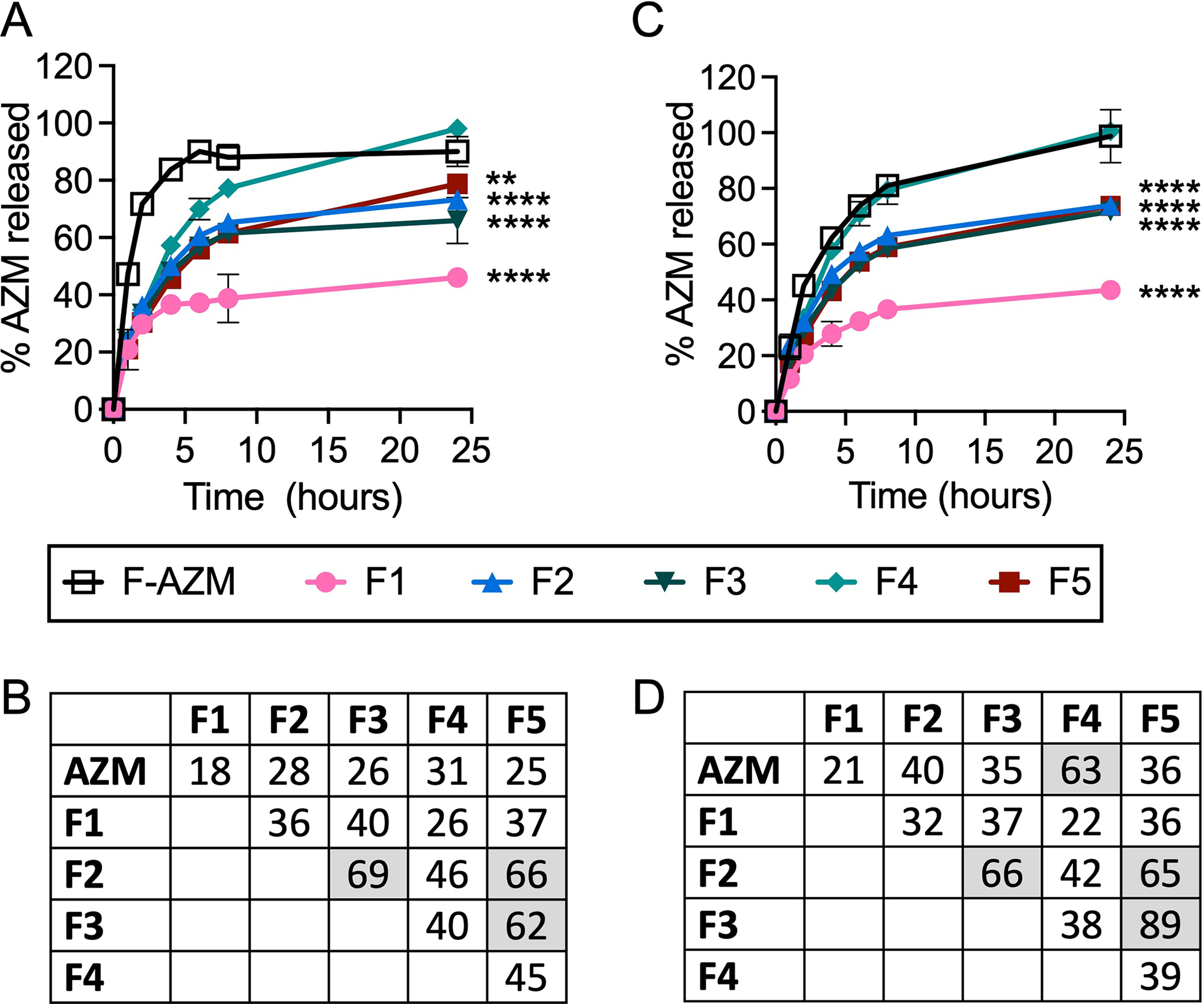
The cumulative release of AZM from each formulation in physiologic conditions. Formulations incubated in snakeskin dialysis tubing against **(A)** PBS buffer or **(C)** 50% FBS in PBS buffer at pH 7.5 at 37°C were quantified for release over 24 h by sampling outside the dialysis tubing to assess the amount of free drug. Similarity factors (*f*_2_) were calculated for each formulation in **(B)** PBS or **(D)** 50% FBS with shaded values *f*_2_ > 50 indicating similarity in release rates. Data represents the average of two experimental replicates with error bars denoting individual measurements. Statistical analysis performed by two-way ANOVA with comparisons relative to free AZM shown for the 24 h timepoint (***p < 0.001, ****p < 0.0001).

Release of AZM from the liposomes was then determined using dialysis and HPLC quantification in both PBS and 50% FBS at 37°C in pH 7.5 (Figure 2). Importantly, AZM measured outside of the dialysis membrane is the fraction that has leaked from the liposome and escaped the dialysis tubing. When using FBS as an incubation media, AZM may also interact with serum proteins prior to escape from the dialysis membrane thereby complicating actual leakage quantification from the liposome. Therefore, we refer to the rate of leakage from the liposome and translocation across the membrane in the absence or presence of serum proteins as the release of AZM. Release of AZM from each formulation was compared to the diffusion of free AZM over 24 h. Nearly all the free AZM is released from the dialysis tubing within 6 h when dialyzed with PBS, and ~50% is released in the first hour (Figure 2A). Importantly, the release rate is reduced in the presence of FBS as AZM can interact with serum proteins prior to release from the dialysis tubing resulting in 50% release in 2 h and 100% release at 24 h (Figure 2C). The difference in release of free AZM from the dialysis tubing in the presence of FBS reaches statistical significance by 1 h (*p* = 0.0001) as determined by Šídák’s multiple comparisons 2-way ANOVA, and the differences persist for up to 6 h. When comparing F-AZM release to the liposomal formulations in PBS (Figure 2A), F4 exhibits continued release to 100% over 24 h, while F2, F3, and F5 perform similarly with 50% leakage achieved in about 4 h and less than 80% release at 24 h. F1 exhibits the slowest release rate over time with only 37% released at 4 h and 46% AZM released at 24 h. Statistical analysis using 2-way ANOVA indicate that all formulations are statistically different from F-AZM as early as 1 h after initiation of the experiment and remain so at 24 h, except F4. Additionally, F2 is not different from F3 and F5 at 24 h, but all other comparisons are found to be different at that same timepoint.

Similar trends are observed when formulations are incubated in FBS resulting in 28% and 44% AZM released at 4 and 24 h for F1, while F2, F3, and F5 achieve ~45% and ~74% at these times, respectively. In FBS, only F1 exhibits statistical difference from free AZM at 1 h (*p* = 0.0006), while the other formulations reach statistical differences at 2 h (F2, F3, F5: *p* < 0.0001; F4: *p* = 0.0005). The statistical significance of each formulation compared to F-AZM at 24 h is denoted in Figure 2C. While F-AZM exhibits a reduced rate of drug release when incubated with FBS, none of the formulations exhibit a difference when incubated in PBS or FBS. Importantly, the formulation with the lowest rate of release (F1) in both experimental conditions is composed of saturated lipids, but also has the lowest EE%. Similarity factors (*f*_2_) were also calculated to compare the release rates of the liposome formulations, which the FDA recommends as a statistical model for comparing tablet dissolution, but have also been used for liposomal formulations (Cipolla et al., 2016). The similarity factors indicate that F2, F3, and F5 are similar in drug release rates in both PBS and FBS (*f*_2_ > 50), and statistically different from free AZM and other formulations (*f*_2_ < 50) (Figures 2B,D), which are in agreement with ANOVA statistical tests. Additionally, F4 exhibits similarity with free AZM when incubated in FBS, but not PBS suggesting a significant impact of serum proteins on the liposomal formulations, which are not observed in PBS.

**FIGURE 3 |:**
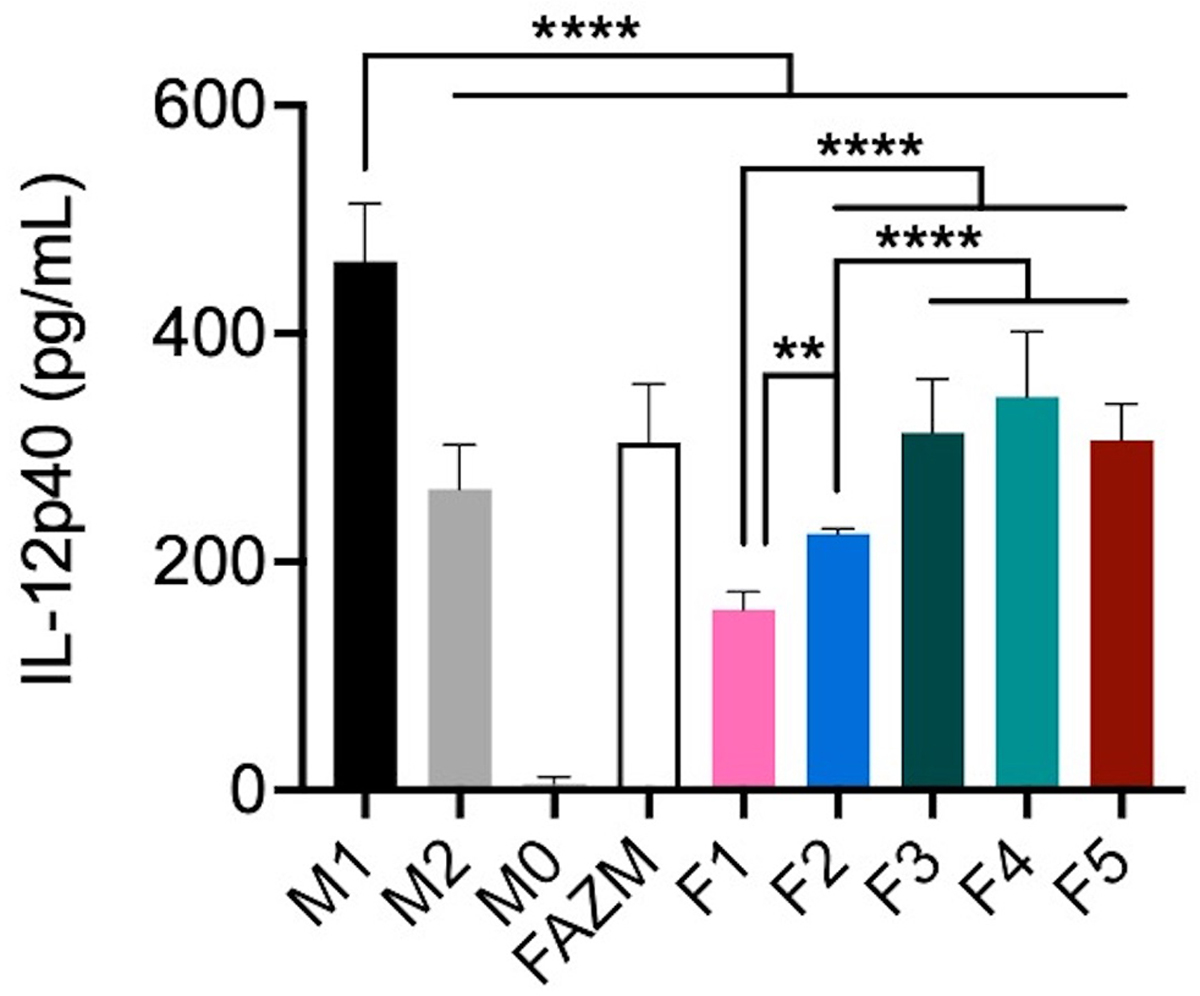
IL-12 protein expression by macrophages using L-AZM formulations. J774 macrophages polarized toward an M1 (IFNγ) or M2 (IL-4/-13) phenotype before being stimulated with LPS, or unstimulated (M0) for use as controls and compared to M1 polarized macrophages treated with 10 μM AZM as free drug (F-AZM) or in liposomal formulations (F1-F5) for 24 h prior to stimulation. Statistical analysis performed by one-way ANOVA with Tukey’s multiple comparisons. (**p < 0.01, ****p < 0.0001).

Based on the differences observed with EE% and drug release rates for each of the formulations, the *in vitro* activity of each formulation was assessed using a standard IL-12 expression assay. IL-12 is a cytokine commonly expressed by pro-inflammatory macrophages in response to stimulation and polarization toward an M1-like phenotype. AZM has previously been shown to reduce the expression of IL-12 in M1 polarized macrophages *in vitro* (Haydar et al., 2019). When AZM is incorporated in liposomes, all formulations exhibit a significant reduction in IL-12 production as compared to M1 controls using one-way ANOVA with Tukey’s multiple comparisons (Figure 3). Additionally, F1 significantly reduces IL-12 levels (*p* < 0.0001) as compared to M2 controls. Only F1 and F2 reduce IL-12 concentrations below those achieved with the same dose of AZM incubated as the free drug (F-AZM; *p* < 0.0001) and F1 and F2 outperform the IL-12 reduction achieved with F3, F4 and F5.

## DISCUSSION

In this study, the design parameters utilized to optimize the lipid composition were based on the differences in lipid saturation, charge, and molar ratios to improve AZM incorporation in the lipid bilayer. Our previous studies utilized a single formulation previously described in the literature, which was developed for its antimicrobial activity (Oh et al., 1995) and repurposed by us for immune cell uptake and polarization to treat the inflamed cardiac tissue after a myocardial infarction (Al-Darraji et al., 2020). Those studies revealed that treating mice with liposomal AZM significantly improved cardiac outcomes and resulted in a 50% reduction in mortality in a murine model of cardiac ischemia (Al-Darraji et al., 2020). The positive outcomes motivated us to identify new formulations with optimized stability for preclinical development. To achieve this, we first examined the stability of a library of formulations and identified five lead candidate formulations that contain AZM which met our criteria based on size and PDI. Our initial formulation remained one of the top candidates for further evaluation in this study (F1) and outperformed the other formulations based on *in vitro* release and macrophage polarization activity, even though EE% ranked last. Collectively, these data indicate a trade-off between encapsulation efficiency and stability when incorporating AZM in the bilayer and positions F1 as the lead candidate formulation for continued preclinical development.

The encapsulation efficiency and stability of lipophilic drugs in a liposomal bilayer are dependent on the intrinsic physico-chemical properties of the drug, including structural motifs and the molecule’s partition coefficient (Log*P*). Liposome studies indicate that hydrophilic molecules with a Log*P* < 1.7 are ideal for aqueous entrapment within the liposome, while molecules with a Log*P* > 5.0 are ideal for incorporation in the lipid bilayer (Gregoriadis and Perrie, 2010). Molecules with an intermediate Log*P* are more likely to partition between the bilayer and the aqueous media. Importantly, AZM exhibits minimal saturation solubility in water (0.07 mg/ml at 25°C) (Arora et al., 2010) and a theoretical Log*P* of 2.7 (Molinspiration Chemoinformatics, https://www.molinspiration.com), presenting the likelihood of AZM to leak from the bilayer into the aqueous media. The rate at which AZM leaks from the bilayer is then regulated by the stability of the interactions between the drug and the lipid bilayer. Liposomal composition can be modified in an effort to optimize the EE% and rate of release from the formulation. The formulation consisting solely of saturated phospholipids (F1) exhibits a modest encapsulation efficiency (~30%), which increases with the inclusion of unsaturated phospholipids within the other liposomes (F2 to F5). Sakai et al., 2008 also found that unsaturated phospholipids can encapsulate more drugs than the saturated counterparts potentially because unsaturated phospholipids remain in a liquid transition phase at room temperature and can create larger vacuoles necessary for drug encapsulation. However, the balance observed between encapsulation efficiency and stability limits the utility of unsaturated lipids for incorporation of AZM in the lipid bilayer. Furthermore, our conclusions are limited by the evaluation of only five formulations and the fact that a minor lipid constituent can have a significant effect on encapsulation efficiency. Nevertheless, a more thorough evaluation of liposomal composition spanning the range of saturated and unsaturated conditions is necessary.

In addition to the saturation of the lipid tails, the anionic phosphate in DSPG serves two critical roles for the stability of the formulations. Firstly, DSPG and other anionic lipids are capable of ion pairing with the cationic charge of AZM to increase the stability of the drug within the formulation. This strategy has been studied previously with AZM using DSPG (Stuhne-Sekalec et al., 1991), cholesteryl hemisuccinate (Ren et al., 2018), and octadecanesulfonate. (Matschiner et al., 1995). Secondly, anionic formulations exhibit greater stability in solution by reducing the likelihood of aggregation as compared to neutrally charged formulations (Zhigaltsev et al., 2002). Anionic liposomal formulations are also more likely to be endocytosed as compared to cationic and neutral lipids (Bajoria et al., 1997), without inducing toxicity observed with cationic lipids. These parameters make DSPG a reasonable candidate for inclusion in a liposome containing AZM. Based on the literature precedent for ion pairing with AZM all formulations had either 25 or 33% DSPG within the formulation and the differences in release rate between formulations suggests that formulation characteristics other than charge are more likely explanations for the differences. While the formulations described here did not specifically focus on differences in charge to promote ion pairing, future studies investigating alternative anionic lipids and different ratios are warranted to further enhance the EE% of AZM in the liposomes.

Regardless of the design parameters leading to the release of AZM from the formulations, the release rate is a critical parameter in the selection of the optimal formulation. Low encapsulation efficiency can be overcome by increasing the number of liposomes in a given dose, and success should be achieved in delivery to the target as long as stability is adequate. Ideally, the formulation would be able to retain the majority of its drug for the duration of its circulation time, but comparing release rates with circulation half-life provides guidance for selection of optimal formulations. PEGylated liposomal formulations are oftentimes exploited for their ability to extend the circulation half-life of a drug by avoiding immune recognition and limiting clearance, which enhances the therapeutic exposure and uptake of the drug within the target tissue. However, when harnessing the phagocytic capacity of monocytes migrating toward the inflamed cardiac tissue, rapid uptake by immune cells is important. Therefore, non-PEGylated liposomes can be strategically employed to improve immune cell uptake and trafficking to the cardiac tissue shortly after administration. Notably, non-PEGylated liposomes injected intravenously exhibit a 4 h half-life in naïve mice (Kierstead et al., 2015), indicating a relatively short window for drug release from the liposome once in circulation. While all of the formulations exhibit drug release over the 24 h experimental period, F1 outperforms the other formulations with only 28% release at 4 h in FBS, and 43% released at 24 h as measured by the non-sequestered AZM. This rate of release is significantly lower than the other formulations and suggests that less than half of the drug would be released over 6 half-lives of the formulation *in vivo*. Importantly, the 4 h half-life of non-PEGylated liposomes was determined in naïve mice and the circulation half-life of non-PEGylated liposomes in mice after induction of cardiac ischemia may be significantly lower since more immune cells are mobilized to respond to the tissue trauma. We have previously shown that <1% of cardiomyocytes in the heart accumulate fluorescently labeled liposomes, and mice do not exhibit signs of cardiac toxicity observed with the same dose of free AZM. (Al-Darraji et al., 2020). However, a complete biodistribution analysis to assess the concentration of drug accumulated in different tissues and among specific cells within the cardiac tissue has not yet been completed. These data, coupled with a pharmacokinetic analysis after inducing a myocardial infarction in mice will provide additional clarity regarding the ideal leakage parameters for L-AZM.

One additional component included in some of the formulations is the fusogenic lipid, DOPE, which promotes an inverted hexagonal lipid structure, while the other lipids in the formulations stabilize the bilayer. Therefore, DOPE should enhance the cytosolic delivery of AZM by promoting fusion between the liposome and endosomal membrane after endocytosis. DOPE has been studied extensively as a helper lipid for gene and drug delivery to enhance cytosolic release (Zhigaltsev et al., 2002; Du et al., 2014). The influence of DOPE on AZM EE% and release rate appear to be minimal when comparing formulations with DOPE (F3, F4, and F5) to those without (F1, F2). While the cytosolic AZM concentration was not directly measured, reduced IL-12 expression was used as an indirect assessment of formulation activity. Notably, all formulations exhibit reduced IL-12 expression as compared to the M1 control, with formulations lacking DOPE (F1 and F2) outperforming the formulations that contain DOPE. IL-12 is a standard cytokine expressed by pro-inflammatory macrophages and serves as a marker for polarization to a pro-inflammatory phenotype. Upon LPS stimulation, IL-12 expression is regulated by NFκB-mediated transcription and directly correlated with p65 translocation from the cytosol into the nucleus, which is inhibited by AZM through an unresolved mechanism (Haydar et al., 2019). While these results may indicate that enhanced cytosolic delivery may not be a predominant factor in the immunomodulatory activity of liposomal AZM delivery, a more robust cellular analysis is needed to explore this potential conclusion.

While the results show promise for future development, there are several limitations associated with the current study including the method for determination of drug release from the formulation, the strategy for removal of free AZM from the liposomes, and the use of IL-12 as a marker for macrophage polarization. First, our goal of determining drug leakage from the formulations were conducted using dialysis tubing in the absence or presence of FBS. In the absence of FBS, release of free AZM from the dialysis compartment provides a baseline rate which accounts for AZM to diffuse across the membrane. When AZM is included in the formulation the rate of release from the dialysis tubing is then based on both the leakage from the liposome and the release from the dialysis tubing. This is further complicated when serum proteins are present, which notably alter the rate of release for the free drug due to the sequestration effect. Therefore, the serum proteins can disrupt the formulations enhancing leakage, but also retaining the AZM within the dialysis tubing leading to a false release rate. However, in this study a comparative assessment was appropriate, and the effect of sequestration are mostly equal across formulations enabling statistical comparisons between formulations. Second, the strategy for removal of free drug prior to dialysis has been performed using several different strategies including size exclusion chromatography, spin filtration, and pressurized filtration chambers. In this study size exclusion chromatography was implemented and the effects of this method on the liposomal formulations cannot be discounted including dilution effects and interactions with the resin. Nonetheless, all formulations were treated equally and the comparisons between formulations remain valid. Third is our use of IL-12 ELISA as a marker of macrophage polarization, which is a standard analyte produced by macrophages indicative of a pro-inflammatory phenotype. IL-12 concentrations have been shown to correlated directly with the extent of polarization across the spectrum of macrophage phenotypes. However, analysis of additional markers of macrophage polarization to fully characterize the macrophage phenotype after treatment with L-AZM is warranted. While outside of the scope of this study, future goals of this work will assess true liposome leakage, compare different purification strategies to remove free AZM, and explore additional markers of macrophage polarization.

Our findings demonstrate a clear difference between the liposome parameters and the biophysical characteristics of each formulation containing AZM. While F1 exhibits only modest encapsulation efficiency, the formulation is bolstered by an optimal size, PDI, charge, release rate, and macrophage polarization activity, which positions this formulation as a lead candidate for continued preclinical development including determination of the pharmacokinetics and biodistribution, which are guided by the liposome stability and leakage studies (Vanić et al., 2019). Additionally, while F1 appears to enhance the immunomodulatory properties of AZM through reduction of IL-12, the exact mechanism by which AZM imparts this functionality is unsolved. Using a two-pronged approach of continued preclinical development of the formulation and elucidation of the mechanism of action of AZM will position L-AZM for significant clinical impact to reduce the burden of disease in patients suffering from an MI.

## Supplementary Material

Supplemental Data

## Figures and Tables

**TABLE 1 |:** Physico-chemical characteristics of five lead liposome formulations.

	Composition	Drug	Diameter (nm)	PDI	Zeta potential (mV)

F1	DSPC:DSPG:Chol (1:1:1)	Empty	73 ± 3	0.20 ± 0.01	−27 ± 4
		AZM	110 ± 6	0.13 ± 0.02	−23 ± 5

F2	POPC:DSPG:Chol (1:1:1)	Empty	72 ± 10	0.27 ± 0.02	−28 ± 4
		AZM	86 ± 5	0.23 ± 0.05	−26 ± 5

F3	POPC:DOPE:DSPG:Chol (1:1:1:1)	Empty	60 ± 4	0.24 ± 0.01	−27 ± 4
		AZM	73 ± 6	0.22 ± 0.01	−24 ± 5

F4	DOPC:DOPE:DSPG:Chol (2:1:2:1)	Empty	66 ± 9	0.29 ± 0.06	−27 ± 4
		AZM	69 ± 8	0.23 ± 0.01	−25 ± 4

F5	DSPC:DOPE:DSPG:Chol (1:1:1:1)	Empty	91 ± 2	0.08 ± 0.02	−24 ± 2
		AZM	111 ± 7	0.09 ± 0.01	−27 ± 4

## Data Availability

The raw data supporting the conclusion of this article will be made available by the authors, without undue reservation.
