# Supplementary material for: Optimization and Characterization of a Liposomal Azithromycin Formulation for Alternative Macrophage Activation
Source: Front Drug Deliv. Author manuscript; Available in PMC 2022 Nov 17. (PMC9670256; doi:10.3389/fddev.2022.908709)
Supplement: Supplemental Data [file NIHMS1849312-supplement-Supplemental_Data.docx]

Supporting information for:

Optimization and characterization of a liposomal azithromycin formulation for alternative macrophage activation

Abdullah A. Masud^1^, Fahd M. Alsharif^1^, Jarrod W. Creameans^1,2^, Jasmine Perdeh^1^, David J. Feola^2^, Vincent J. Venditto^1*^

^1^ University of Kentucky, College of Pharmacy, Department of Pharmaceutical Sciences, Lexington, KY, USA

^2^ University of Kentucky, College of Pharmacy, Department of Pharmacy Practice and Science, Lexington, KY, USA

*** Correspondence:**Vincent J. Venditto, PhD
Vincent.venditto@uky.edu

Keywords: azithromycin, drug delivery, immune modulation, liposome, macrophage

**Supplementary Table 1.** Preliminary characterization of liposomal formulations for lead formulation studies.

|  | Composition | Ratio | Lipid Conc. | EE% | Z avg | PDI |
| --- | --- | --- | --- | --- | --- | --- |
| 1 | DSPC:DSPG:CHOL | 1:1:1 | 40 mM | 40 +/- 9 | 149-153 | 0.10-0.15 |
| 2 | DSPC:DSPG:CHOL | 3:3:1 | 40 mM | 62 +/- 2 | 350-625 | 0.66-0.78 |
| 3 | DSPC:DOPE:DSPG:Chol | 1:1:1:1 | 40 mM | 66 +/- 4 | 122-125 | 0.14-0.16 |
| 4 | DSPC:DOPE:DSPG:Chol | 2:1:2:1 | 40 mM | 70 +/- 6 | 160-164 | 0.42-0.48 |
| 5 | DOPC:DSPG:Chol | 1:1:1 | 40 mM | 80 +/- 1 | 341-414 | 0.56-0.72 |
| 6 | DOPC:DSPG:Chol | 3:3:1 | 40 mM | 76 +/- 3 | 396-455 | 0.55-0.86 |
| 7 | DOPC:DOPE:DSPG:Chol | 1:1:1:1 | 40 mM | 49 +/- 11 | 61-62 | 0.23-0.24 |
| 8 | DOPC:DOPE:DSPG:Chol | 2:1:2:1 | 40 mM | 76 +/- 4 | 129-313 | 0.41-0.45 |
| 9 | POPC:DSPG:Chol | 1:1:1 | 40 mM | 82 +/- 4 | 121-124 | 0.30-0.35 |
| 10 | POPC:DSPG:Chol | 3:3:1 | 40 mM | 78 +/- 11 | 157-162 | 0.55-0.59 |
| 11 | POPC:DOPE:DSPG:Chol | 1:1:1:1 | 40 mM | 59 +/- 6 | 83-86 | 0.27-0.32 |
| 12 | POPC:DOPE:DSPG:Chol | 2:1:2:1 | 40 mM | 58 +/- 10 | 215-310 | 0.40-0.82 |
|  |  |  |  |  |  |  |
|  | Composition | Ratio | Lipid Conc. | EE% | Z avg | PDI |
| 13 | DSPC:DSPG:CHOL | 1:1:1 | 60 mM | 65 +/- 3 | 134-137 | 0.16-0.20 |
| 14 | DSPC:DSPG:CHOL | 3:3:1 | 60 mM | 31 +/- 13 | 627-724 | 0.48-0.86 |
| 15 | DSPC:DOPE:DSPG:Chol | 1:1:1:1 | 60 mM | 48 +/- 11 | 119-121 | 0.14-0.16 |
| 16 | DSPC:DOPE:DSPG:Chol | 2:1:2:1 | 60 mM | 57 +/- 9 | 186-193 | 0.41-0.46 |
| 17 | DOPC:DSPG:Chol | 1:1:1 | 60 mM | 82 +/- 7 | 183-194 | 0.68-0.77 |
| 18 | DOPC:DSPG:Chol | 3:3:1 | 60 mM | 44 +/- 9 | 343-378 | 0.96-1.00 |
| 19 | DOPC:DOPE:DSPG:Chol | 1:1:1:1 | 60 mM | 25 +/- 12 | 96-99 | 0.23-0.24 |
| 20 | DOPC:DOPE:DSPG:Chol | 2:1:2:1 | 60 mM | 38 +/- 7 | 126-129 | 0.39-0.45 |
| 21 | POPC:DSPG:Chol | 1:1:1 | 60 mM | 72 +/- 9 | 100-102 | 0.32-0.33 |
| 22 | POPC:DSPG:Chol | 3:3:1 | 60 mM | 66 +/- 4 | 181-191 | 0.67-0.70 |
| 23 | POPC:DOPE:DSPG:Chol | 1:1:1:1 | 60 mM | 57 +/- 6 | 87-88 | 0.20-0.23 |
| 24 | POPC:DOPE:DSPG:Chol | 2:1:2:1 | 60 mM | 46 +/- 6 | 202-226 | 0.68-0.73 |

**Supplementary Table 2**. Encapsulation efficiency of formulation with 10 mol% and 30 mol% AZM, based on phospholipid content, added to the thin film prior to hydration.

|  | **F1** | **F2** | **F3** | **F4** | **F5** |
| --- | --- | --- | --- | --- | --- |
| **10 mol%** | 30.0 ± 0.5 | 61.7 ± 3.3 | 66.6 ± 2.0 | 61.0 ± 2.6 | 38.5 ± 1.1 |
| **30 mol%** | 48.5 ± 5.9 | 90.3 ± 7.4 | 84.7 ± 9.0 | 79.6 ± 7.0 | 69.4 ± 2.2 |

**Supplementary Table 3**. Calculated difference factors (f1) comparing free azithromycin and each formulation in PBS (A) and FBS (B). Shaded values denote similar release rates.

| f1 | F1 | F2 | F3 | F4 | F5 |
| --- | --- | --- | --- | --- | --- |
| AZM | 56 | 34 | 39 | 27 | 38 |
| F1 |  | 48 | 38 | 71 | 40 |
| F2 |  |  | 7 | 18 | 9 |
| F3 |  |  |  | 20 | 6 |
| F4 |  |  |  |  | 22 |

| f1 | F1 | F2 | F3 | F4 | F5 |
| --- | --- | --- | --- | --- | --- |
| AZM | 45 | 18 | 23 | 5 | 23 |
| F1 |  | 61 | 49 | 91 | 48 |
| F2 |  |  | 8 | 23 | 8 |
| F3 |  |  |  | 24 | 2 |
| F4 |  |  |  |  | 30 |

A B
